# Pulmonary Atelectasis After Sedation With Propofol vs Propofol-Ketamine for Magnetic Resonance Imaging in Children: A Randomized Clinical Trial

**DOI:** 10.1001/jamanetworkopen.2024.33029

**Published:** 2024-11-01

**Authors:** Yu Jeong Bang, Jeayoun Kim, Nam-Su Gil, Woo Seog Sim, Hyun Joo Ahn, Mi Hye Park, Sangmin Maria Lee, Dong-Jae Kim, Ji Seon Jeong

**Affiliations:** 1 Department of Anesthesiology and Pain Medicine, Samsung Medical Center, Sungkyunkwan University School of Medicine, Seoul, Korea

## Abstract

**Importance:**

Little is known about the impact of different anesthetic agents used for routine magnetic resonance imaging (MRI) sedation on pulmonary function in children.

**Objective:**

To compare the incidence of pulmonary atelectasis after MRI sedation with propofol vs propofol-ketamine.

**Design, Setting, and Participants:**

This double-masked randomized clinical trial screened 117 consecutive pediatric patients aged 3 to 12 years with American Society of Anesthesiologists physical status I to II undergoing elective MRI under deep sedation from November 2, 2022, to April 28, 2023, at a tertiary referral center. Four patients met the exclusion criteria, and 5 patients refused to participate. The participants and outcome assessors were masked to the group allocation.

**Interventions:**

During the MRI, the propofol group received 0.2 mL/kg of 1% propofol and 2 mL of 0.9% saline followed by a continuous infusion of propofol (200 μg/kg/min) and 0.9% saline (0.04 mL/kg/min). The propofol-ketamine group received 0.2 mL/kg of 0.5% propofol and 1 mg/kg of ketamine followed by a continuous infusion of propofol (100 μg/kg/min) and ketamine (20 μg/kg/min).

**Main Outcome and Measure:**

The incidence of atelectasis assessed by lung ultrasonography examination.

**Results:**

A total of 107 children (median [IQR] age, 5 [4-6] years; 62 male [57.9%]), with 54 in the propofol group and 53 in the propofol-ketamine group, were analyzed in this study. Notably, 48 (88.9%) and 31 (58.5%) patients had atelectasis in the propofol and propofol-ketamine groups, respectively (relative risk, 0.7; 95% CI, 0.5-0.8; *P* < .001). The incidence of desaturation and interruption of the MRI due to airway intervention or spontaneous movement did not significantly differ between the groups. The propofol-ketamine group showed a faster emergence time than the propofol group (15 [9-23] vs 25 [22-27] minutes in the propofol-ketamine vs propofol group; median difference in time, 9.0 minutes; 95% CI, 6.0-12.0 minutes; *P* < .001). No patient was withdrawn from the trial due to adverse effects.

**Conclusions and Relevance:**

In this randomized clinical trial, the propofol-ketamine combination reduced sedation-induced atelectasis while allowing for faster emergence compared with propofol alone.

**Trial Registration:**

cris.nih.go.kr Identifier: KCT0007699

## Key Points

**Question:**

What is the incidence of atelectasis among children sedated for magnetic resonance imaging with propofol vs propofol-ketamine?

**Findings:**

In this randomized clinical trial of 107 pediatric patients, the incidence of pulmonary atelectasis and the total lung score, which was evaluated by lung ultrasonography, were significantly lower in the propofol-ketamine sedation group compared with the propofol sedation group.

**Meaning:**

In pediatric patients undergoing magnetic resonance imaging under deep sedation, the propofol-ketamine combination reduced the frequency and severity of sedation-induced atelectasis while maintaining effective sedation and allowing a faster emergence time.

## Teaser

This randomized clinical trial of pediatric patients examines whether the combination of propofol and ketamine during magnetic resonance imaging sedation reduces the incidence of atelectasis compared with propofol infusion alone.

## Introduction

The demand for pediatric sedation during magnetic resonance imaging (MRI) continues to increase.^1,2^ Although MRI itself is not a painful procedure, it requires relatively deep sedation to ensure immobility during image acquisition.^2^ Achieving an adequate level of sedation while ensuring safety is key to the success of MRI. However, little is known about the effect of different anesthetic agents on pulmonary function during sedation.

Although a single sedative is generally considered safer than a combination of multiple sedatives,^3^ the concomitant administration of sedatives with different mechanisms offers certain advantages. Concomitant administration maximizes the benefit of individual drugs and decreases the dosage of each drug, thus minimizing the risk of adverse effects.^4,5^ Propofol, which has a fast onset and rapid recovery time, is mainly used for sedation during pediatric MRI.^2,6^ However, even at subhypnotic doses, propofol is associated with collapse of the upper airway in a dose-dependent manner.^7^ On the other hand, ketamine has the advantage of maintaining the tone of the upper airway and preserving spontaneous breathing in response to upper airway obstruction.^8^ Maintaining airway patency could be easier when propofol and ketamine are used in combination than with propofol alone; therefore, propofol and ketamine are sometimes used in combination.^9^

Pulmonary atelectasis occurs in approximately 80% of pediatric patients undergoing MRI using propofol sedation.^10^ Specifically, the deep sedation and supine position required for pediatric MRI are known to influence the occurrence of atelectasis. Persistent atelectasis impairs arterial blood oxygenation, causing hypoxemia during and after anesthesia.^11,12^ Additionally, it is considered as a contributing mechanism of pulmonary complications, including pneumonia.^13,14^ To date, no previous studies have explored the effect of sedatives on atelectasis and its clinical implications. We hypothesized that the combination of propofol and ketamine during pediatric MRI sedation would reduce the incidence of atelectasis compared with propofol infusion alone. Therefore, this study aimed to compare the incidence of atelectasis after pediatric MRI sedation using the propofol-ketamine combination vs propofol alone. We also compared the quality of sedation, periprocedural adverse events, and recovery profiles between the groups.

## Methods

This study is a single-center, parallel, double-masked randomized clinical trial with a 1:1 concealed allocation conducted at the Samsung Medical Center, a tertiary referral hospital in Seoul, Korea. The study protocol was approved by the Samsung Medical Center Institutional Review Board on August 23, 2022, and prospectively registered at the Clinical Research Information Service before recruitment of the first participant. Written informed consent was obtained from parents or legal guardians before participation in the study. The children were also provided with an age-tailored brochure for the intervention. The study was conducted in accordance with Good Clinical Practice guidelines and followed the Declaration of Helsinki^15^ guidelines. The study protocol, including a prespecified statistical plan for the trial, is available in Supplement 1. The study followed the Consolidated Standards of Reporting Trials (CONSORT) reporting guideline.

### Participants

The inclusion criteria were children aged 3 to 12 years with American Society of Anesthesiologists physical status I to II who were undergoing elective 3T MRI under deep sedation. The exclusion criteria were history of thoracic surgery and lung disease, respiratory infection, airway abnormality, increased intracranial or intraocular pressure, uncontrolled hypertension, uncontrolled seizure, and allergy or contraindications for the study drugs. Patients with pulmonary pathology (atelectasis, pneumonia, pneumothorax, or pleural effusion) based on preoperative chest radiographs were also excluded.

### Randomization and Masking

The participants were randomly divided into 2 groups: a propofol group (control) and a propofol-ketamine group (intervention). A study assistant who otherwise had no role in this study prepared sequentially numbered sealed envelopes using random sheets generated by a web-based program with a block size of 4. Allocation sequence was concealed from the researcher (J.K.) who recruited the participants. The anesthesia nurse opened the envelope and prepared the study drug in a separate drug preparation space; this nurse had no other role in this study. The study drugs were labeled as A (propofol) and B (0.9% saline or ketamine) and set in an infusion pump system (SpaceStation MRI; B. Braun Medical), which was shielded in aluminum housing. All of the study drug was administered to the patients using the equivalent volume and delivery rate. The participants and their legal guardians, MRI technicians, radiologists, attending nurses in the postanesthesia care unit (PACU), attending anesthesiologists, and outcome accessors were masked to the drugs.

### Sedation Protocol

The sedation process was standardized according to the institutional protocol. All patients underwent chest radiography within 7 days before the MRI. In the pediatric sedation clinic, all children underwent physical examinations, including noninvasive blood pressure, heart rate, oxygen saturation, and tympanic body temperature and were evaluated for signs of respiratory infection. Premedication using 4 μg/kg of glycopyrrolate was performed intravenously 3 minutes before entering the MRI suite. In the MRI suite, standard monitoring, including electrocardiography, noninvasive blood pressure, oxygen saturation, and end-tidal carbon dioxide, was performed. Blood pressure was measured every 5 minutes, and other vital signs were continuously monitored using an MRI-compatible monitoring device (IRadimed 3880; IRadimed Corp).

Sedatives were administered according to the group as follows. In the propofol group, 0.2 mL/kg of 1% propofol and 2 mL of 0.9% saline (placebo) were administered, followed by continuous infusion of propofol and 0.9% saline at a rate of 200 μg/kg/min and of 0.04 mL/kg/min, respectively. In the propofol-ketamine group, 0.2 mL/kg of 0.5% propofol (mixture of 1% propofol and 0.9% saline) and 1 mg/kg of diluted ketamine in 0.9% saline (total 2 mL) were administered followed by continuous infusion of propofol and ketamine at a rate of 100 and 20 μg/kg/min, respectively. We determined the dosage of sedatives based on previous research^16,17^ and our clinical experience.

If sedation was not induced in either group, the patients were administered an additional 1 mg/kg of propofol every 1 minute until they became unconscious. After an appropriate sedation level was confirmed, MRI was initiated with the patient in the neck-extension position using a shoulder roll. A Modified Observer’s Alertness/Sedation Scale was used to confirm sedation level, and a score of 2 or lower indicated an appropriate sedation level. If coughing, snoring, or movement interrupted the acquisition of diagnostic images, the scanning processes were stopped to eliminate interrupting factors. If a mean blood pressure lower than 50 mm Hg did not recover within 10 minutes or was accompanied with bradycardia, we used an inotropic agent to increase blood pressure at the clinician’s discretion.

### Lung and Diaphragm Ultrasonography

**Figure 1.**
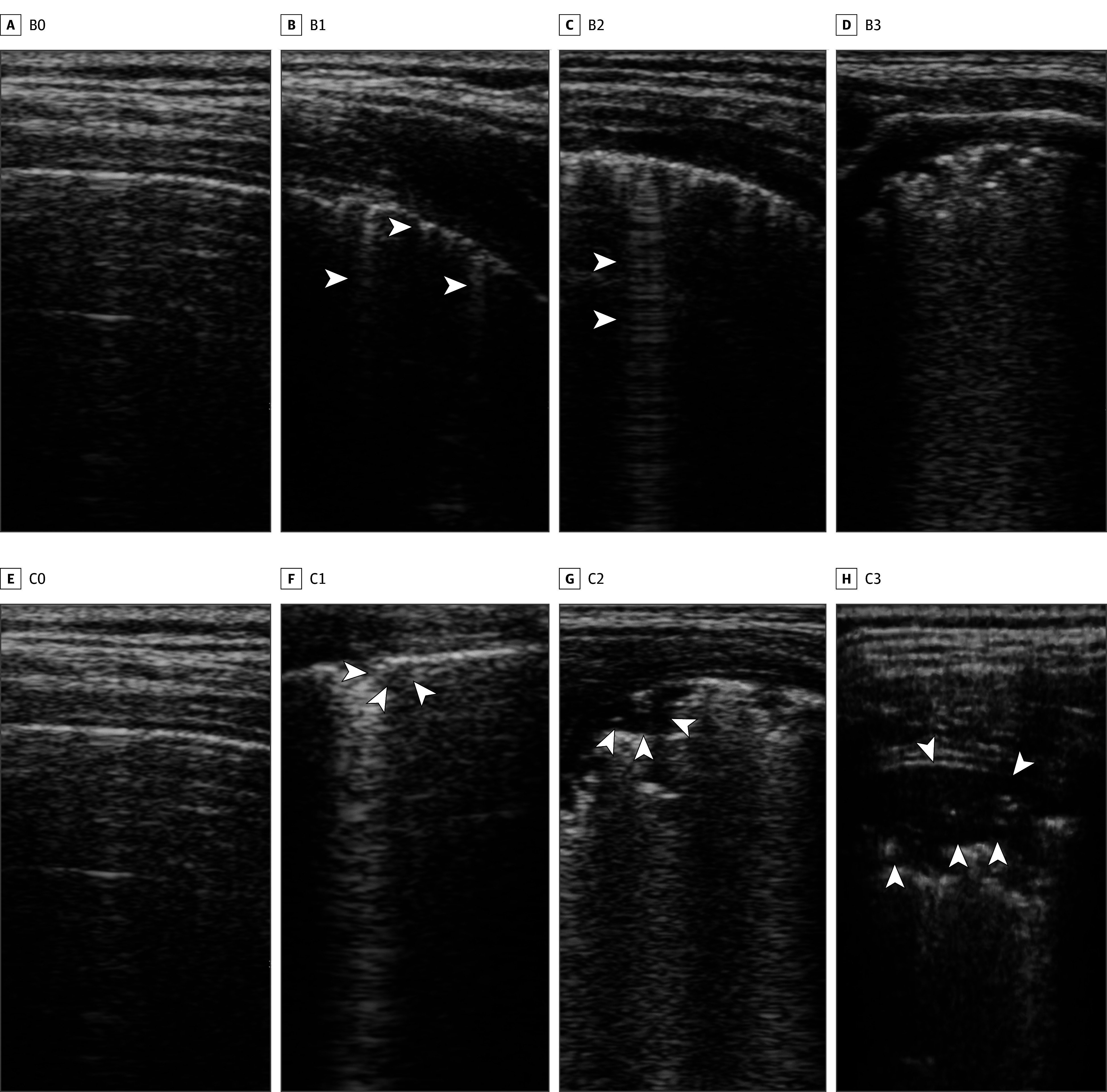
Lung Ultrasound Findings of Consolidations and B-Lines The degree of B-lines was divided into 4 grades as follows: B0, fewer than 3 isolated B-lines; B1, multiple well-defined B-lines; B2, multiple coalescent B-lines; and B3, white lung. The degree of consolidation was divided into 4 grades as follows: C0, no consolidation; C1, minimal juxtapleural consolidations; C2, small-sized consolidations; and C3, large-sized consolidations. Arrowheads indicate pathologic findings.

Patients in both groups were transferred to the PACU immediately after the MRI, and a lung ultrasonography examination was performed when they were still sedated. All ultrasonography examinations were performed by 2 experienced, masked investigators (Y.J.B. and J.K.) using a handheld ultrasonography system (VScan Air; GE Healthcare) at a selected depth of 4 cm. Six regions in each hemithorax were scanned in all participants following the methods of a previous study^18^ by dividing anterior, lateral, and posterior zones (separated by the anterior and posterior axillary lines) into the upper (1 cm above the nipples) and lower (above the diaphragm) portions. In addition, the posterior caudal regions were assessed using an intercostal posterobasal view. The patients were placed in a supine position for the anterior and lateral section scans and in a lateral decubitus position for the posterior section scan. All patients underwent 14 predefined standard scans, and 10-second video clips for each scan were simultaneously recorded. Thereafter, 2 anesthesiologists (Y.J.B. and J.K.) independently evaluated the video clips of the lung ultrasonography following the scoring system described by Song et al (Figure 1).^19^ The degree of juxtapleural consolidation (C score) was graded from 0 to 3 as follows: 0, no consolidation; 1, minimal consolidation; 2, small consolidation; and 3, large consolidation. The degree of B-lines (B score) was graded from 0 to 3 as follows: 0, fewer than 3 isolated B-lines; 1, multiple B-lines; 2, multiple coalescent B-lines; and 3, white lungs. In cases involving different decisions, the final decision was drawn through a joint review. Atelectasis was considered if the juxtapleural consolidation score (C score) was greater than 1 on more than 1 region based on the lung ultrasonography examination. The total lung score was calculated as the sum of the C and B scores.

Additionally, bilateral diaphragms were scanned to evaluate excursions during spontaneous breathing.^20^ Diaphragmatic excursion was defined as the perpendicular distance between the upper border of the liver or spleen at the end of expiration and inspiration. The diaphragm excursion values were presented as the mean excursion depth on each side.

### Outcomes

The primary outcome was the incidence of lung atelectasis on lung ultrasonography on arrival at the PACU. Secondary outcomes included total lung score, diaphragm excursion, parasternal muscle thickness on arrival at the PACU, the induction dose of propofol for sedation, image quality, adverse events during sedation and recovery, the recovery profile (time to emergence and duration of PACU stay), nurse satisfaction score for manageability, and parent-reported outcomes within 24 hours after sedation. Baseline data included patient characteristics, comorbidities and diagnoses, and type of MRI. Image quality was rated by an MRI technologist or radiologist (1, not acceptable at all; 2, diagnosis impossible; 3, single protocol not acceptable; 4, acceptable; and 5, very good). Adverse events during sedation were evaluated as hypertension (an increase of >20% from baseline mean blood pressure), hypotension (a decrease of >20% from baseline mean blood pressure), tachycardia (an increase of >20% from baseline heart rate), bradycardia (a decrease of >20% from baseline heart rate), desaturation (oxygen saturation <95%), airway intervention, movement events, and interruption of MRI. Adverse events during the recovery phase included nausea, vomiting, dizziness, and emergence delirium during the PACU stay. Emergence delirium was considered at a score of 10 or higher on the Pediatric Anesthesia Emergence Delirium Scale.^21^ Furthermore, parent-reported outcomes were collected. At discharge, parents were educated to monitor their children’s body temperature and the presence of any adverse outcomes after discharge, which included dizziness, drowsiness, and respiratory complications. The investigators performed telephone interviews with the parents 24 hours later to collect data and asked them to rate their satisfaction with the sedation process and recovery. Regarding respiratory complications, the data on the presence of temperature greater than 38 °C, cough, sputum, and other abnormal signs were collected. Parasternal muscle thickness was not reported because the change during the inspiratory cycle was too minimal to measure accurately in pediatric patients.

### Statistical Analysis

Sample size was calculated based on clinical data from a previous study,^22^ which reported that atelectasis was developed in 82% of children who underwent MRI under propofol sedation. We expected the incidence of atelectasis in the propofol-ketamine group to decrease by 35% compared with the propofol group. The required sample size was 47, with a power of 85% and an α = .05. Assuming a dropout rate of 10%, the study sample size was set at a total of 108 participants, with 54 participants in each group.

For continuous variables, medians (IQRs) or means (SDs) were presented as appropriate based on normality assumption tested by the Shapiro-Wilk test. Group comparison was performed using a 2-sample *t* test or Mann-Whitney test as appropriate for continuous variables. All differences in medians and their corresponding 95% CIs were computed using Hodges-Lehmann estimates with asymptotic SE. Categorical data were presented as numbers (percentages) and compared using the χ^2^ test or Fisher exact test, as appropriate. In addition, we performed multivariable logistic regressions to adjust for the effect of imbalanced variables. A 2-sided *P* < .05 was considered statistically significant. All statistical analyses were performed using MedCalc, version 19.5.6 (MedCalc Software Ltd) and SPSS software, version 27.0 (IBM Inc).

## Results

**Figure 2.**
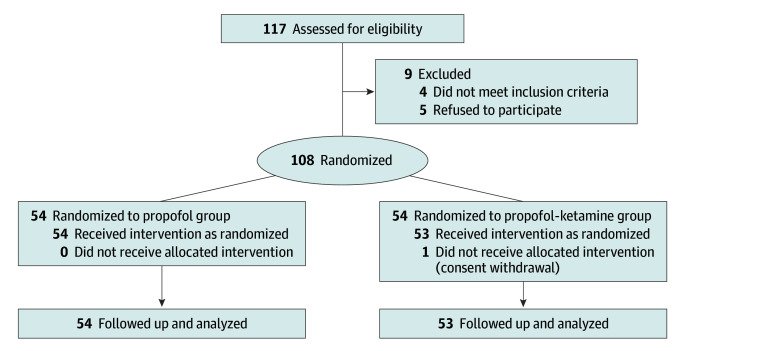
Consolidated Standards of Reporting Trials Flow Diagram of Patients Included in the Study

A total of 117 children who underwent MRI under deep sedation from November 2, 2022, to April 28, 2023, were assessed for eligibility. Of these, 5 patients did not provide consent, and 4 were excluded due to upper respiratory tract infection. Thus, 108 patients were enrolled and randomly assigned to either the propofol (54 patients) or propofol-ketamine (54 patients) group. One patient in the propofol-ketamine group withdrew consent, resulting in 107 patients (median [IQR] age, 5 [4-6] years; 62 male [57.9%] and 45 female [42.1%]) being included in the primary analysis (Figure 2). The parents of 2 patients in the propofol group and 3 patients in the propofol-ketamine group did not respond to the telephone interview 24 hours after sedation, resulting in missing values for parent-reported outcomes. The demographic data and baseline characteristics were comparable between the 2 groups except for height (Table 1).

**Table 1.** Demographic Data and Baseline Characteristics of the Participants^a^

Variable	Propofol group (n = 54)	Propofol-ketamine group (n = 53)
Age, median (IQR), y	5 (4-6)	5 (4-5)
Height, median (IQR), cm	110.6 (102.2-121.1)	105.1 (96.6-110.8)
Weight, median (IQR), kg	19.4 (16-24)	18 (14.9-20.1)
BMI, median (IQR)	16.1 (15.2-16.9)	16.5 (15.1-17.8)
Sex		
Male	30 (55.6)	32 (60.4)
Female	24 (44.4)	21 (39.6)
ASA physical status		
I	18 (33.3)	15 (28.3)
II	36 (66.7)	38 (71.7)
Type of MRI		
Head	45 (83.3)	38 (71.7)
Lung and mediastinum	5 (9.3)	2 (3.8)
Whole spine	1 (1.9)	6 (11.3)
Whole body	1 (1.9)	5 (9.4)
Other	2 (3.7)	2 (3.8)
Time of scanning, median (IQR), min	34 (28-40)	34 (27-38)

Abbreviations: ASA, American Society of Anesthesiologists; BMI, body mass index (calculated as weight in kilograms divided by height in meters squared); MRI, magnetic resonance imaging.

^a^ Data are presented as number (percentage) unless otherwise indicated.

Notably, 48 (88.9%) and 31 (58.5%) patients were confirmed to have atelectasis using lung ultrasonography in the propofol and propofol–ketamine groups, respectively (relative risk, 0.7; 95% CI, 0.5-0.8; *P* < .001) (Table 2). After adjustment for the effect of imbalanced height between the 2 groups, a logistic regression analysis revealed that the propofol group had a higher incidence of atelectasis than the propofol-ketamine group (odds ratio, 0.2; 95% CI, 0.1-0.5; *P* = .001). Furthermore, the total lung score was significantly higher in the propofol group (median [IQR], 6 [3-10]) compared with the propofol-ketamine group (median [IQR], 2 [0-4]; median difference, 4.0; 95% CI, 2.0-5.0; *P* < .001). In addition, both B and C scores were significantly higher in the propofol group than in the propofol-ketamine group (median [IQR] B scores, 2 [0-4] vs 0 [0-0]; median difference, 1.0 [95% CI, 0.0-2.0]; *P* = .001; median [IQR] C scores, 4 [1-6] vs 1 [0-2]; median difference, 2.0 [1.0-3.0]; *P* < .001). However, no significant difference was observed in diaphragm excursion between the groups.

**Table 2.** Lung Ultrasound Scores and Diaphragm Excursion

Variable	Propofol group (n = 54)	Propofol-ketamine group (n = 53)	Relative risk (95% CI) or median difference (95% CI)^a^	*P* value
Atelectasis, No. (%)	48 (88.9)	31 (58.5)	0.7 (0.5 to 0.8)	<.001
Total lung score^b^ (range, 0-84), median (IQR)	6 (3 to 10)	2 (0 to 4)	4.0 (2.0 to 5.0)	<.001
B-line (B) score^c^ (range, 0-42), median (IQR)	2 (0 to 4)	0 (0 to 0)	1.0 (0.0 to 2.0)	.001
Consolidation (C) score^d^ (range, 0-42), median (IQR)	4 (1 to 6)	1 (0 to 2)	2.0 (1.0 to 3.0)	<.001
Diaphragm excursion, mean (SD), mm	6.09 (2.33)	5.89 (2.25)	0.2 (−0.7 to 1.1)	.66

^a^ Continuous variables are reported as median or mean difference (95% CI), whereas categorical variables are reported as relative risk (95% CI).

^b^ The total lung score was calculated as the sum of the C and B scores.

^c^ The degree of B-lines (B score) was graded from 0 to 3 as follows: 0, fewer than 3 isolated B-lines; 1, multiple B-lines; 2, multiple coalescent B-lines; and 3, white lungs.

^d^ The degree of juxtapleural consolidation (C score) was graded from 0 to 3 as follows: 0, no consolidation; 1, minimal consolidation; 2, small consolidation; and 3, large consolidation.

The safety profiles during sedation are given in Table 3. During the sedation, hypertension was more frequent in the propofol-ketamine group (5 [9.3%] vs 18 [34.0%]; *P* = .002), whereas hypotension was more frequent in the propofol group (38 [70.4%] vs 24 [45.3%]; *P* = .009). However, hypertension was transient, and none of the patients required treatment. In contrast, the median (IQR) duration of hypotension was 25 (15-35) minutes and 12 (7.5-23.5) minutes in the propofol and propofol-ketamine groups, respectively (median difference, 10 minutes; 95% CI, 4-17 minutes; *P* = .002). In particular, 1 patient in the propofol group who presented with severe hypotension required medication, with a decrease of approximately 60% compared with baseline blood pressure (37/19 mm Hg). The incidence of desaturation was similar between groups. Interruptions in MRI due to airway intervention or spontaneous movement and the quality of the images was also comparable between the groups.

**Table 3.** Sedation and Recovery Profiles and Adverse Events

Variable	Propofol group	Propofol-ketamine group	Relative risk (95% CI) or median difference (95% CI)	*P* value
**During MRI **				
No. of patients	54	53	NA	NA
Sedation duration, median (IQR), min	37 (30 to 43)	36 (29 to 41)	1.0 (−3.0 to 4.0)	.66
Anesthetic administration				
Induction dose of propofol, median (IQR), mg/kg	2.7 (2.0 to 3.6)	1.0 (1.0 to 1.0)	1.1 (1.0 to 1.8)	<.001
Additional boluses to sedate, No.				
0	22	48	NA	<.001
1	18	5		
2	9	0		
3	4	0		
4	1	0		
Propofol, median (IQR), mg/kg/min	0.31 (0.27 to 0.35)	0.17 (0.14 to 0.22)	0.1 (0.1 to 0.2)	<.001
Ketamine, mean (SD), mg/kg/min	NA	0.05 (0.02)	NA	NA
Adverse events, No. (%)				
Tachycardia	4 (7.4)	6 (11.3)	1.5 (0.5 to 5.1)	.53
Bradycardia	4 (7.4)	3 (5.7)	0.8 (0.2 to 3.3)	>.99
Hypertension	5 (9.3)	18 (34.0)	3.7 (1.5 to 9.2)	.002
Hypotension	38 (70.4)	24 (45.3)	0.6 (0.5 to 0.9)	.009
Desaturation	2 (3.7)	2 (3.8)	1.0 (0.1 to 7.0)	>.99
Airway intervention	4 (7.4)	1 (1.9)	0.3 (0.0 to 2.2)	.18
Movement event	4 (7.4)	10 (18.9)	2.5 (0.9 to 7.6)	.08
Interruption of MRI	7 (13.0)	8 (15.1)	1.2 (0.5 to 3.0)	.75
Miscellaneous	1 (1.9)	0	0.3 (0.0 to 8.2)	>.99
Quality of MRI, median (IQR) (score, 1-5)^a^	5 (4 to 5)	5 (5 to 5)	0.0 (0.0 to 0.0)	.26
**During PACU stay**				
No. of patients	54	53	NA	NA
PACU stay, median (IQR), min	30 (30 to 32)	30 (30 to 30)	0.0 (0.0 to 0.0)	.02
Time to emergence, median (IQR), min	25 (22 to 27)	15 (9 to 23)	9.0 (6.0 to 12.0)	<.001
Emergence delirium, No. (%)	10 (18.5)	6 (11.3)	0.6 (0.2 to 1.6)	.32
Nurse satisfaction score, median (IQR) (range, 0-10)^b^	9 (8 to 9)	10 (9 to 10)	−1.0 (−1.0 to 0.0)	.006
Adverse events, No. (%)	7 (13.0)	10 (18.9)	1.5 (0.6 to 3.5)	.40
Dizziness	5 (9.3)	6 (11.3)	1.2 (0.4 to 3.8)	.70
Nausea	1 (1.9)	3 (5.7)	3.1 (0.3 to 28.5)	.35
Vomiting	0	0	NA	NA
Others	1 (1.9)	1 (1.9)	1.0 (0.1 to 16.2)	>.99
**Within 24 h after sedation^c^**				
Patients who did not respond to telephone calls, No.	52	50	NA	NA
Parent satisfaction score^d^				
1	0	0	NA	.01
2	0	0		
3	7	0		
4	5	10		
5	40	50		
Adverse events, No. (%)	26 (50.0)	15 (30.0)	0.6 (0.4 to 1.0)	.04
Dizziness	10 (19.2)	10 (20.0)	1.0 (0.5 to 2.3)	.92
Drowsiness	11 (21.2)	6 (12.0)	0.6 (0.2 to 1.4)	.22
Respiratory complications	13 (25.0)	1 (2.0)	0.1 (0.0 to 0.6)	.001
Fever	10	0	0.0 (0.0 to 0.8)	.04
Cough	7	1	0.1 (0.0 to 1.2)	.07
Sputum	1	0	0.1 (0.0 to 2.2)	.15
Other	0 (0.0)	1 (2.0)	3.1 (0.1 to 73.4)	.49

Abbreviations: MRI, magnetic resonance imaging; NA, not applicable; PACU, postanesthesia care unit.

^a^ The MRI quality was evaluated using a 5-point Likert scale (1, not acceptable at all; 2, diagnosis impossible; 3, single protocol not acceptable; 4, acceptable; and 5, very good).

^b^ Nurse satisfaction was evaluated using an 11- point numeric rating scale (with 0 indicating extremely difficult to manage to 10 indicating extremely easy to manage).

^c^ Denominators that do not equal the sample sizes are due to missing data.

^d^ Parent satisfaction was evaluated using a 5-point Likert scale (1, very dissatisfied; 2, somewhat dissatisfied; 3, neutral; 4, somewhat satisfied; and 5, very satisfied).

Patients in the propofol-ketamine group showed faster emergence in the PACU and higher nurse and parent satisfaction than in the propofol group (Table 3). Notably, respiratory complications after discharge, including fever, cough, and sputum, were more frequent in the propofol group, whereas the incidences of other complications, such as dizziness and drowsiness, were similar in both groups. One participant in the propofol-ketamine group showed disorientation that was resolved spontaneously within 6 hours.

## Discussion

This randomized clinical trial demonstrated that the propofol-ketamine combination reduced pulmonary atelectasis after sedation, offering a more stable cardiovascular and improved recovery profile than propofol alone in children undergoing deep sedation for MRI. Specifically, the propofol-ketamine combination was associated with a lower incidence of pulmonary atelectasis than propofol alone for pediatric MRI sedation (approximately a 1.5-fold decrease). Although sedation with spontaneous breathing is considered safer than general anesthesia,^23^ atelectasis during sedation is frequently reported.^22,24,25^ During sedation for pediatric MRI, atelectasis develops as a result of decreased lung elastic recoil, which results in a closing capacity that exceeds functional residual capacity.^10,22^ Pulmonary atelectasis occurred more frequently with propofol alone, which possibly contributed to respiratory complications, including fever, cough, and sputum production. Atelectasis impairs blood oxygenation and can progress to hypoxemia.^13^ It can also induce local tissue inflammation and immune dysfunction associated with the development of pneumonia and potential lung injury.^14,26,27^ However, in general cases, atelectasis has a benign clinical course and resolves spontaneously. Similarly, in this study, although the incidence of atelectasis was higher in the propofol group and this group had more frequent fever, cough, and sputum after discharge, most cases were self-limited, and airway interventions required during the sedation and desaturation events were similar between the 2 groups. Most children underwent the procedure uneventfully, and it is unclear whether procedural atelectasis significantly affects the patient clinical course in otherwise healthy children. However, our results suggest that the propofol-ketamine combination may offer potential benefits to vulnerable pediatric patients at increased risk for respiratory complications.

Various mechanisms, including absorption atelectasis, tissue compression, and decreased surfactant function, have been proposed to explain the development of anesthesia-induced atelectasis.^28,29^ One possible explanation for absorption atelectasis is shallow breathing induced by anesthetics administration. The use of anesthetics causes a loss of muscle tone and a decrease in functional residual capacity, leading to absorption atelectasis.^14,29,30^ Another potential mechanism underlying absorption atelectasis is upper airway obstruction. Even at subhypnotic doses, propofol is associated with an increase in upper airway collapsibility in a dose-dependent manner in healthy volunteers,^7^ whereas ketamine maintains upper airway patency and elicits a compensatory respiratory response.^8^ Although atelectasis occurred frequently in the propofol group, we did not find any differences in diaphragmatic excursion between both groups. This finding suggests that the occurrence of atelectasis may be attributed to differences in upper airway patency rather than to differences in shallow breathing. However, in our study, we could not confirm whether atelectasis was due to upper airway obstruction because the degree of obstruction was not measured.

A previous study revealed that, compared with propofol alone, the propofol-ketamine combination results in more frequent spontaneous movements, which prolonged the scanning process.^31^ In the current study, although movement events were more frequently observed in the propofol-ketamine group compared with the propofol group, this difference was not statistically significant. In addition, because most movements were limited to the fingers or toes of the children in the propofol-ketamine group, these did not necessarily lead to the interruption of MRI. This inconsistency may be attributed to the method of sedative administration. In a previous study,^31^ ketamine was administered only at the time of induction, and continuous infusion of ketamine was not performed.

Propofol reduces mean arterial blood pressure primarily due to vasodilation and has a depressant effect on the myocardium.^32,33,34^ Although ketamine also has negative inotropic effects, its sympathomimetic effect achieved by inhibiting the reuptake of norepinephrine mitigates the hypotensive effects of propofol.^35^ In the current study, 1 patient in the propofol group experienced severe hypotension, requiring medication for a decrease of approximately 60% from baseline blood pressure (37/19 mm Hg). Additionally, hypotensive events were more frequent in the propofol group compared with the propofol-ketamine group, which is consistent with previous findings.^36,37^ However, both the propofol and propofol-ketamine groups exhibited a high incidence of hypotensive events and atelectasis in this study. The high incidence of atelectasis and hypotension may be attributed to the high doses of sedatives required for deep sedation during MRI procedures. These findings underscore the importance for clinicians to carefully weigh the benefits of achieving a successful MRI in 1 attempt against the potential risks to hemodynamic stability and the development of atelectasis.

Propofol-ketamine combination therapy led to significantly shorter emergence times, and the satisfaction of nurses in the PACU was better with this combination. Consistent with the results of a previous study,^31^ our results showed that the recovery time (time to emergence) of the propofol-ketamine combination was shorter than that of propofol alone, presumably because of the propofol-saving effect with a reduction in additional boluses and the continuous infusion rate of propofol. The rapid onset and offset of the effect are additional advantages of the propofol-ketamine combination in outpatient clinic settings. However, the difference in nurse satisfaction and time of PACU stay was not clinically significant, suggesting that both sedation regimens facilitated quick recovery and were easily manageable during the recovery phase.

### Limitations

Our study has limitations. First, as a single-center study of Korean children with American Society of Anesthesiologists physical status I to II, the generalizability of our findings may be limited. Additionally, the age range of participants was from 3 to 12 years, which encompasses a broad developmental spectrum. Although there can be significant differences in body size and anesthetic effects within this age range, most of our patients were preschool-aged (3-6 years). This distribution helps maintain the reliability of our findings despite the broad age range. Future studies should consider a narrower age range to control for these variables more effectively. Second, we could not compare the changes in the lung ultrasonography images before and after anesthetic administration because we did not perform an ultrasonography before patient sedation. Although baseline lung ultrasonograms could not be obtained due to poor cooperation by conscious pediatric patients, children with abnormal findings suggestive of lung disease were excluded through chest radiographs and physical examination before enrollment. Third, we did not evaluate upper airway obstruction, which is a major cause of atelectasis. Because we could not access the children or assess the degree of airway obstruction in them during MRI, data on airway patency were not collected. Fourth, the 1-day outcomes were collected via telephone interviews. We educated the parents to observe any symptoms of respiratory complications and residual sedative effects and followed up after 24 hours by telephone. Some cases of mild complications may possibly have been neglected and not collected. Moreover, follow-up only until 24 hours limited the data regarding delayed-onset or long-term complications.

## Conclusions

This randomized clinical trial found that the propofol-ketamine combination significantly reduced postsedation atelectasis, allowing a faster emergence time, compared with propofol alone in pediatric patients undergoing deep sedation for MRI. Additional studies are needed to determine the clinical relevance of reducing sedation-induced atelectasis on the patient’s clinical course.

## Notes

Supplement 1.
Trial Protocol

Supplement 2.
Data Sharing Statement

## References

[1] Deen J, Vandevivere Y, Van de Putte P. Challenges in the anesthetic management of ambulatory patients in the MRI suites. Curr Opin Anaesthesiol. 2017;30(6):670-675. doi:10.1097/ACO.0000000000000513 28817401

[2] Mallory MD, Travers C, Cravero JP, Kamat PP, Tsze D, Hertzog JH. Pediatric sedation/anesthesia for MRI: results from the Pediatric Sedation Research Consortium. J Magn Reson Imaging. 2023;57(4):1106-1113. doi:10.1002/jmri.28392 36173243

[3] Khurmi N, Patel P, Kraus M, Trentman T. Pharmacologic considerations for pediatric sedation and anesthesia outside the operating room: a review for anesthesia and non-anesthesia providers. Paediatr Drugs. 2017;19(5):435-446. doi:10.1007/s40272-017-0241-5 28597354

[4] Iqbal AU, Shuster ME, Baum CR. Ketofol for procedural sedation and analgesia in the pediatric population. Pediatr Emerg Care. 2022;38(1):28-33. doi:10.1097/PEC.0000000000002599 34986578

[5] Alletag MJ, Auerbach MA, Baum CR. Ketamine, propofol, and ketofol use for pediatric sedation. Pediatr Emerg Care. 2012;28(12):1391-1395. doi:10.1097/PEC.0b013e318276fde2 23222112

[6] Kamat PP, McCracken CE, Simon HK, . Trends in outpatient procedural sedation: 2007-2018. Pediatrics. 2020;145(5):e20193559. doi:10.1542/peds.2019-3559 32332053

[7] Sundman E, Witt H, Sandin R, . Pharyngeal function and airway protection during subhypnotic concentrations of propofol, isoflurane, and sevoflurane: volunteers examined by pharyngeal videoradiography and simultaneous manometry. Anesthesiology. 2001;95(5):1125-1132. doi:10.1097/00000542-200111000-00016 11684981

[8] Mishima G, Sanuki T, Sato S, Kobayashi M, Kurata S, Ayuse T. Upper-airway collapsibility and compensatory responses under moderate sedation with ketamine, dexmedetomidine, and propofol in healthy volunteers. Physiol Rep. 2020;8(10):e14439. doi:10.14814/phy2.14439 32441458 PMC7243198

[9] Grunwell JR, Travers C, Stormorken AG, . Pediatric procedural sedation using the combination of ketamine and propofol outside of the emergency department: a report from the Pediatric Sedation Research Consortium. Pediatr Crit Care Med. 2017;18(8):e356-e363. doi:10.1097/PCC.0000000000001246 28650904 PMC6287759

[10] Riva T, Pascolo F, Huber M, . Evaluation of atelectasis using electrical impedance tomography during procedural deep sedation for MRI in small children: a prospective observational trial. J Clin Anesth. 2022;77:110626. doi:10.1016/j.jclinane.2021.110626 34902800

[11] Motoyama EK, Glazener CH. Hypoxemia after general anesthesia in children. Anesth Analg. 1986;65(3):267-272. doi:10.1213/00000539-198603000-00008 3954092

[12] Xue FS, Huang YG, Tong SY, . A comparative study of early postoperative hypoxemia in infants, children, and adults undergoing elective plastic surgery. Anesth Analg. 1996;83(4):709-715. doi:10.1213/00000539-199610000-00008 8831307

[13] Zeng C, Lagier D, Lee JW, Vidal Melo MF. Perioperative pulmonary atelectasis, part I: biology and mechanisms. Anesthesiology. 2022;136(1):181-205. doi:10.1097/ALN.0000000000003943 34499087 PMC9869183

[14] Lagier D, Zeng C, Fernandez-Bustamante A, Vidal Melo MF; Clinical Implications. Perioperative pulmonary atelectasis, part II. Anesthesiology. 2022;136(1):206-236. doi:10.1097/ALN.0000000000004009 34710217 PMC9885487

[15] World Medical Association. World Medical Association Declaration of Helsinki: ethical principles for medical research involving human subjects. JAMA. 2013;310(20):2191-2194. doi:10.1001/jama.2013.281053 24141714

[16] Usher AG, Kearney RA, Tsui BC. Propofol total intravenous anesthesia for MRI in children. Paediatr Anaesth. 2005;15(1):23-28. doi:10.1111/j.1460-9592.2004.01390.x 15649159

[17] Tosun Z, Akin A, Guler G, Esmaoglu A, Boyaci A. Dexmedetomidine-ketamine and propofol-ketamine combinations for anesthesia in spontaneously breathing pediatric patients undergoing cardiac catheterization. J Cardiothorac Vasc Anesth. 2006;20(4):515-519. doi:10.1053/j.jvca.2005.07.018 16884981

[18] Acosta CM, Maidana GA, Jacovitti D, . Accuracy of transthoracic lung ultrasound for diagnosing anesthesia-induced atelectasis in children. Anesthesiology. 2014;120(6):1370-1379. doi:10.1097/ALN.0000000000000231 24662376

[19] Song IK, Jang YE, Lee JH, . Effect of different fraction of inspired oxygen on development of atelectasis in mechanically ventilated children: a randomized controlled trial. Paediatr Anaesth. 2019;29(10):1033-1039. doi:10.1111/pan.13718 31411351

[20] El-Halaby H, Abdel-Hady H, Alsawah G, Abdelrahman A, El-Tahan H. Sonographic evaluation of diaphragmatic excursion and thickness in healthy infants and children. J Ultrasound Med. 2016;35(1):167-175. doi:10.7863/ultra.15.01082 26679203

[21] Sikich N, Lerman J. Development and psychometric evaluation of the pediatric anesthesia emergence delirium scale. Anesthesiology. 2004;100(5):1138-1145. doi:10.1097/00000542-200405000-00015 15114210

[22] Lutterbey G, Wattjes MP, Doerr D, Fischer NJ, Gieseke J Jr, Schild HH. Atelectasis in children undergoing either propofol infusion or positive pressure ventilation anesthesia for magnetic resonance imaging. Paediatr Anaesth. 2007;17(2):121-125. doi:10.1111/j.1460-9592.2006.02045.x 17238882

[23] Brinjikji W, Murad MH, Rabinstein AA, Cloft HJ, Lanzino G, Kallmes DF. Conscious sedation versus general anesthesia during endovascular acute ischemic stroke treatment: a systematic review and meta-analysis. AJNR Am J Neuroradiol. 2015;36(3):525-529. doi:10.3174/ajnr.A4159 25395655 PMC8013063

[24] Choe JW, Jung SW, Song JK, . Predictive factors of atelectasis following endoscopic resection. Dig Dis Sci. 2016;61(1):181-188. doi:10.1007/s10620-015-3844-0 26289260

[25] Sargent MA, McEachern AM, Jamieson DH, Kahwaji R. Atelectasis on pediatric chest CT: comparison of sedation techniques. Pediatr Radiol. 1999;29(7):509-513. doi:10.1007/s002470050632 10398785

[26] Restrepo RD, Braverman J. Current challenges in the recognition, prevention and treatment of perioperative pulmonary atelectasis. Expert Rev Respir Med. 2015;9(1):97-107. doi:10.1586/17476348.2015.996134 25541220

[27] Ko E, Yoo KY, Lim CH, Jun S, Lee K, Kim YH. Is atelectasis related to the development of postoperative pneumonia? a retrospective single center study. BMC Anesthesiol. 2023;23(1):77. doi:10.1186/s12871-023-02020-4 36906539 PMC10007747

[28] Kim PH, Park YS, Yoon HM, . Factors associated with occurrence of atelectasis during sedation for imaging in pediatric patients: a retrospective single center cohort study. J Clin Med. 2021;10(16):3598. doi:10.3390/jcm10163598 34441894 PMC8397091

[29] Hedenstierna G, Edmark L. Mechanisms of atelectasis in the perioperative period. Best Pract Res Clin Anaesthesiol. 2010;24(2):157-169. doi:10.1016/j.bpa.2009.12.002 20608554

[30] Janssen ML, Jonkman AH, Wennen M, Wils EJ, Endeman H, Heunks L. Diaphragm excursions as proxy for tidal volume during spontaneous breathing in invasively ventilated ICU patients. Intensive Care Med Exp. 2023;11(1):73. doi:10.1186/s40635-023-00553-z 37891413 PMC10611662

[31] Schmitz A, Weiss M, Kellenberger C, . Sedation for magnetic resonance imaging using propofol with or without ketamine at induction in pediatrics—a prospective randomized double-blinded study. Paediatr Anaesth. 2018;28(3):264-274. doi:10.1111/pan.13315 29377404

[32] Pagel PS, Warltier DC. Negative inotropic effects of propofol as evaluated by the regional preload recruitable stroke work relationship in chronically instrumented dogs. Anesthesiology. 1993;78(1):100-108. doi:10.1097/00000542-199301000-00015 8424542

[33] Robinson BJ, Ebert TJ, O’Brien TJ, Colinco MD, Muzi M. Mechanisms whereby propofol mediates peripheral vasodilation in humans: sympathoinhibition or direct vascular relaxation? Anesthesiology. 1997;86(1):64-72. doi:10.1097/00000542-199701000-00010 9009941

[34] Gelissen HP, Epema AH, Henning RH, Krijnen HJ, Hennis PJ, den Hertog A. Inotropic effects of propofol, thiopental, midazolam, etomidate, and ketamine on isolated human atrial muscle. Anesthesiology. 1996;84(2):397-403. doi:10.1097/00000542-199602000-00019 8602672

[35] Lippmann M, Appel PL, Mok MS, Shoemaker WC. Sequential cardiorespiratory patterns of anesthetic induction with ketamine in critically ill patients. Crit Care Med. 1983;11(9):730-734. doi:10.1097/00003246-198309000-00012 6884053

[36] Jalili M, Bahreini M, Doosti-Irani A, Masoomi R, Arbab M, Mirfazaelian H. Ketamine-propofol combination (ketofol) vs propofol for procedural sedation and analgesia: systematic review and meta-analysis. Am J Emerg Med. 2016;34(3):558-569. doi:10.1016/j.ajem.2015.12.074 26809929

[37] Hayes JA, Aljuhani T, De Oliveira K, Johnston BC. Safety and efficacy of the combination of propofol and ketamine for procedural sedation/anesthesia in the pediatric population: a systematic review and meta-analysis. Anesth Analg. 2021;132(4):979-992. doi:10.1213/ANE.0000000000004967 32665470

