# Supplementary material for: Pulmonary Atelectasis After Sedation With Propofol vs Propofol-Ketamine for Magnetic Resonance Imaging in Children: A Randomized Clinical Trial
Source: JAMA Netw Open. 2024 Nov 1;7(11):e2433029. doi: 10.1001/jamanetworkopen.2024.33029 (PMC11530935; doi:10.1001/jamanetworkopen.2024.33029)
Supplement: Supplement 1. — Trial Protocol [file jamanetwopen-e2433029-s001.pdf]

Pulmonary Atelectasis Following Sedation for Magnetic Resonance Imaging in Children Using Propofol Alone versus Propofol–Ketamine Combination

# : A Randomized Controlled Trial

Original version – Study Protocol

Version 1.0

August 15, 2022

Principle Investigator:

Ji Seon Jeong, MD, PhD

Samsung Medical Center, Sungkyunkwan University School of Medicine

Organization and Sponsor:

Samsung Medical Center

Investigating Centers:

Samsung Medical Center

Clinical Trials Identifier:

KCT0007699, https://cris.nih.go.kr

Signature Page for Investigator

Pulmonary Atelectasis Following Sedation for Magnetic Resonance Imaging in Children Using Propofol Alone versus Propofol–Ketamine Combination: A Randomized Controlled Trial

Protocol Version: 1.0

Version Date: August 15, 2022

I have read this protocol and agree to conduct this protocol in accordance with all stipulations of the protocol and accordance with Good Clinical Practice (GCP) and relevant local regulatory requirements that govern the conduct of clinical research.

Ji Seon Jeong		August 15, 2022
Principal Investigator Name	Signature	Date

Table of Contents

1. List of Abbreviations and Relevant Definitions ... 1
2. Protocol Summary ... 2
3. Introduction and Hypothesis ... 5
   3.1. Introduction ... 5
   3.2. Hypothesis ... 6
4. Study outcomes and definition ... 6
   4.1. Primary outcome ... 6
   4.2. Secondary outcomes ... 7
   4.3. Safety outcomes ... 9
5. Study population ... 10
   5.1. Subject selection ... 10
   5.2. Inclusion criteria ... 10
   5.3. Exclusion criteria ... 12
   5.4. Exit (drop out) from the Trial ... 12
6. Study design ... 12
   6.1. Study Overview ... 12
   6.2. Screening, Randomization and Blinding Procedure ... 13
      6.2.1. Screening ... 13
      6.2.2. Randomization ... 14
      6.2.3. Blinding ... 14
   6.3. Study Intervention ... 15
      6.3.1. Description of the Study Intervention ... 15
      6.3.2. Duration of Intervention ... 16
      6.3.3. Anesthesia, Surgery, and Postoperative Management ... 16
      6.4.1. Baseline Characteristics ... 17
      6.4.2. Procedure Characteristics ... 17
      6.4.3. Postoperative variables (outcome variables) ... 17
7. Data Management ... 18
   7.1. Data Handling and Record Keeping ... 18

7.2. Quality Control and Quality Assurance 18
8. Adverse Events 19
8.1 Definitions 19
8.2. Reporting of SAEs 19
9. Ethical and Regulatory Considerations 20
9.1. Informed Consent 20
9.2. Independent Ethics Committee Approval 20
9.3. Responsibilities of the Investigator(s) 21
10. Statistical Analysis Plan 21
10.1. Randomization & allocation concealment 21
10.2. Data collection/management 22
6.4. Data collection and Follow-up .... **Error! Bookmark not defined.**
10.3. Statistical Hypothesis 22
10.3.1. The primary statistical hypothesis is as follows: 22
10.3.2. The secondary statistical hypothesis is as follows: 22
10.4. Sample Size Calculation 23
10.5. Statistical Methods 23
10.5.1. Primary outcome 24
10.5.2. Secondary outcomes 24
10.5.3. Baseline characteristics 24
11. Funding 24
12. Schedule of Events 25
13. Timeline 25
14. Trial Participants 26
14.1. Participating Site 26
14.2. Principal investigator and research physician 26
14.4 Investigators 26
14.5. End-point adjudication committee 27
14.6. Data monitoring committee 27
14.8. Statistical experts 27
15. Reference 28

List of Abbreviations and Relevant Definitions

ASA	American Society of Anesthesiologists
CPAP	Continuous positive airway pressure
CRF	Case record form
C score	Consolidation score
GCP	Good Clinical Practice
IRB	Institutional review boards
MRI	Magnetic Resonance Imaging
PACU	Post-anesthesia care unit
SAE	Severe adverse events

1. Protocol Summary

**Title**	Pulmonary Atelectasis Following Sedation for Magnetic Resonance Imaging in Children Using Propofol Alone versus Propofol–Ketamine Combination: A Randomized Controlled Trial
**Principal Investigator**	Ji Seon Jeong
**Funding**	Samsung Medical Center
**Background**	Achieving an adequate deep sedation level while ensuring safety is key to the success of Magnetic Resonance Imaging (MRI) scans in pediatric patients. Deep sedation can lead to a decrease in lung elastic recoil and a closing volume that exceeds functional residual capacity, resulting in pulmonary atelectasis. In fact, approximately 80% of pediatric MRI patients who receive using propofol sedation develop pulmonary atelectasis. While most cases resolve spontaneously, there is a risk of progression to hypoxemia and pneumonia.<br>The question of which anesthetics are the most effective and safe for achieving deep sedation during pediatric MRI scans remains uncertain. Propofol is mainly used for sedation during pediatric MRI scans due to its rapid onset and quick recovery time. However, even at subhypnotic doses, propofol is associated with upper airway collapse in a dose-dependent manner. On the other hand, ketamine has the advantage of maintaining upper airway collapsibility and a compensatory respiratory response. However, its could be less effective because of occasional random movements which is characteristic of dissociative sedation.<br>While a single sedative is generally considered safer than a combination of multiple sedatives, the concomitant administration of sedatives with different mechanisms offers certain advantages. It maximizes the benefit of individual drugs and decrease the dosage of each drug, thus minimizing the risk of adverse effects.<br>We hypothesized that the combination of propofol and ketamine during

	pediatric MRI sedation would decrease the incidence of atelectasis compared with propofol infusion alone. Therefore, this study aimed to compare the incidence of atelectasis following pediatric MRI sedation when using the propofol–ketamine combination versus propofol alone.
**Hypothesis**	The combination of propofol and ketamine during pediatric MRI sedation would reduce the incidence of atelectasis compared with propofol infusion alone.
**Primary Endpoint**	**Outcome: the incidence of pulmonary atelectasis determined by lung ultrasonography#**<br>Time-point: Upon arrival at the post-anesthesia care unit (PACU)<br>Definition: Atelectasis is defined as a juxtapleural consolidation score (C score) greater than 1 in more than one region, as determined by the lung ultrasound examination.[13]
**Secondary Endpoints**	**1. Outcome: Total lung score#**<br>Time-point: Upon arrival at the PACU<br>**2. Outcome: Diaphragm excursion***<br>Time-point: Upon arrival at the PACU<br>**3. Outcome: Respiratory complications**<br>Time-point: Within 24 hours after MRI sedation.<br>**4. Outcome: Image quality**<br>Time-point: At the end of MRI sedation.<br>#Total lung score and atelectasis will be evaluated by following scoring system described by Song et al. (Fig. 1) [13].<br>*Diaphragm excursion will be evaluated during spontaneous breathing according to the previous research [14].
**Study Population**	Children aged 3 to 12 years with American Society of Anesthesiologists physical status I and II undergoing elective MRI scans under deep sedation.
**Sample Size**	N=108
**Study Design**	Study Type: Interventional Study<br>Study Purpose: Prevention<br>Intervention Model: Parallel

	Blinding/Masking: Double<br>Blinded Subjects: Patients, Outcome Accessor<br>Allocation: Randomized controlled trial
**Accrual Period**	Six months of recruitment and follow up
**Study Duration**	From institutional review board approval to 6 month (approximately June 2023)
**Study Intervention**	Before anesthesia induction, study participants will be randomized in a 1:1 manner to one of the following anesthetic:<br>• Propofol group: Participants will receive 0.2 mL/kg of 1% propofol and 2 mL of 0.9% saline (placebo) followed by a continuous infusion of propofol at a rate of 200 μg/kg/min and 0.9% saline at a rate of 0.04 mL/kg/min.<br>• The propofol–ketamine group: Participants will receive 0.2 mL/kg of 0.5% propofol (mixture of 1% propofol and 0.9% saline) and 1 mg/kg of diluted ketamine in 0.9% saline (total 2 mL), followed by a continuous infusion of propofol at a rate of 100 μg/kg/min and ketamine at a rate of 20 μg/kg/min.
**Assessments**	Both groups will be followed from the day of MRI to 24 hours after MRI sedation.

3. Introduction and Hypothesis

3.1. Introduction

The demand for pediatric sedation during magnetic resonance imaging (MRI) is increasing to relieve anxiety and ensure image quality by minimizing movement during the procedure.[1,2] Although MRI itself is not a painful procedure, achieving a sufficient level of deep sedation is necessary to ensure immobility during image acquisition.[2] The success of MRI scans in pediatric patients depends on achieving this deep sedation level while ensuring safety.

Deep sedation can lead to decreased lung elastic recoil and a closing volume that exceeds functional residual capacity, potentially resulting in pulmonary atelectasis. In fact, pulmonary atelectasis occurs in approximately 80% of pediatric MRI patients who receive propofol sedation. While most cases resolve spontaneously, there is a risk of progression to hypoxemia and pneumonia, posing a potential injury risk and necessitating additional medical resources.[3,4]

Currently there is no international consensus regarding the most effective and safe for achieving for achieving deep sedation during pediatric MRI scans. Propofol is mainly used for sedation during pediatric MRI scans due to its rapid onset and quick recovery time. However, even at subhypnotic doses, propofol is associated with upper airway collapse in a dose-dependent manner. On the other hands, ketamine has the advantage of preseving both upper airway collapsibility and a compensatory respiratory response, however, it could be less effective due to occasional random movements typically associated with dissociative sedation.

Although controversial, retrospective data suggest that using a single sedative is generally considered safer than a combination of multiple sedatives.[5] However, the concomitant administration of sedatives with different mechanisms offers certain advantages. It maximizes the benefit of individual drugs and decrease the dosage of each drug, thereby minimizing the risk of adverse effects.

We hypothesize that the combination of propofol and ketamine during pediatric MRI sedation would decrease the incidence of atelectasis compared with propofol infusion alone. Therefore, this study aims to compare the incidence of atelectasis following pediatric MRI sedation

between the propofol–ketamine combination versus propofol alone.

3.2. Hypothesis

We hypothesize that the combination of propofol and ketamine during pediatric MRI sedation would decrease the incidence of atelectasis compared with propofol infusion alone.

4. Study outcomes and definition

4.1. Primary outcome

The primary outcome of the study is the incidence of lung atelectasis on lung ultrasonography.

• Time-points: upon arrival at the post-anesthesia care unit (PACU)

• Definition

- Atelectasis: Atelectasis will be defined as a juxtapleural consolidation score (C score) exceeding 1 in more than one region based on the lung ultrasound examination.
- Two anesthesiologists will independently evaluate the video clips of the lung ultrasound following the scoring system described by Song et al.[6]
- Six regions in each hemithorax will be scanned in all participants following the methodology from a previous study[7] by dividing anterior, lateral, and posterior zones (separated by the anterior and posterior axillary lines) into the upper (1 cm above the nipples) and lower portions (above the diaphragm). In addition, the posterior caudal regions will be assessed using an intercostal posterobasal view.
- The degree of juxtapleural consolidation (C score) will be graded from 0 to 3 as follows[6]:

**C score**			
0	1	2	3
no consolidation	minimal consolidation	small consolidation	large consolidation

- Confusing cases will be adjudicated by a joint review, using stored image scan of ultrasonography and clinical evidence, by investigators who are unaware of patients' random assignments.

4.2. Secondary outcomes

The secondary end point of the study will be as follows

1) <u>Total lung score:</u>

• Time-points: upon arrival at PACU

• Definition: The total lung score is defined as the sum of the C and B scores of 14 lung region of bilateral hemithorax.

- Two anesthesiologists will independently evaluate the video clips of the lung ultrasound following the scoring system described by Song et al.[6]
- Six regions in each hemithorax will be scanned in all participants following the methodology from a previous study[7] by dividing anterior, lateral, and posterior zones (separated by the anterior and posterior axillary lines) into the upper (1 cm above the nipples) and lower portions (above the diaphragm). In addition, the posterior caudal regions will be assessed using an intercostal posterobasal view.
- Lung score of each region is defined by the sum of the C and B scores.

**C score**			
0	1	2	3
no consolidation	minimal consolidation	small consolidation	large consolidation
**B score**			
0	1	2	3
<3 isolated B lines	multiple B lines	multiple coalescent B lines	white lungs

2) Diaphragm excursion:

- Time-points: upon arrival at PACU
- Definition: The perpendicular distance between the upper border of the liver or spleen at the end of expiration and inspiration.[8] The diaphragm excursion values will be presented as the average excursion depth on each side.

3) Movement event and interruption for scanning process

- Time-points: During the sedation for MRI
- Definition: Movement event is defined as any movement after the induction of anesthesia during the MRI procedure. If coughing, snoring, or movement interrupted the acquisition of diagnostic images, the scanning processes will be paused to eliminate interrupting factors.

4) Quality of MRI scan

- Time-points: At the end of MRI scan.
- Definition: A radiologist performing the MRI scan of study subjects will score the quality of MRI scan using a 5-point Likert scale (1, very dissatisfied; 2, somewhat dissatisfied; 3, neutral; 4, somewhat satisfied; and 5, very satisfied).

5) Time to emergence

- Definition: The time duration from the end of sedation to the eye-opening.

6) Duration of PACU stay

- Definition: The time duration from the end of sedation to discharge from PACU

7) Emergence delirium

- Time-points: during the PACU stay
- Definition: Emergence delirium is defined as a score of ≥10 on the Pediatric Anesthesia Emergence Delirium Scale.[9]

8) Nurse satisfaction rated from 0 (very unsatisfied) to 10 (very satisfied)

• Time-points: during the PACU stay

• Definition: An attending nurse in the PACU will evaluate the quality of recovery using a 0 to 10 numeric score.

9) Parent satisfaction

• Time-points: from the start of sedation until 24 hours after sedation

• Definition: Parent satisfaction will be evaluated using a 5-point Likert scale (1, very dissatisfied; 2, somewhat dissatisfied; 3, neutral; 4, somewhat satisfied; and 5, very satisfied).

10) Respiratory complications

• Time-points: within 24 hours after MRI sedation

• Definition: Respiratory complications are defined as fever >38 ℃, cough, or the presence of sputum.

- The one-day outcomes will be collected through telephone interviews. The investigators will educate the parents to observe any symptoms of respiratory complications and residual sedative effects and follow up after 24 h.

4.3. Safety outcomes

The safety outcomes of this study will be as follows

1) Tachycardia
   • Time-points: During the MRI sedation.

   • Definition: An increase of > 20% from baseline heart rate
2) Bracydarcia
   • Time-points: During the MRI sedation.

   • Definition: A decrease of > 20% from baseline heart rate

3) Hypertension

- Time-points: During the MRI sedation.

- Definition: An increase of >20% from baseline mean blood pressure

4) Hypotension

- Time-points: During the MRI sedation.

- Definition: A decrease of >20% from baseline mean blood pressure

5) Desaturation

- Time-points: During the MRI sedation.

- Definition: Pulse oximetry value < 95%

6) Airway intervention

- Time-points: During the MRI sedation.

- Definition: Airway intervention is defined as any of the following procedures to treat sedation-induced respiratory depression: mild prodding, jaw thrust, reduction of study drug infusion rate, Guedel airway insertion, bag-mask-assisted ventilation, or intubation.

7) Dizziness and nausea

- Time-points: During the PACU stay

5. Study population

5.1. Subject selection

This study can only fulfill its objectives if appropriate subjects are enrolled. In addition to the eligibility criteria listed below, all relevant medical and non-medical factors will be considered when determining the enrollment of individual subject.

5.2. Inclusion criteria

- Pediatric patients aged 3 to 12 years old
- Patients with an American Society of Anesthesiologists (ASA) physical status I or II

- Patients undergoing elective MRI scans under deep sedation

ASA physical status[10]

ASA PS classification	Definition	Adult examples, including, but not limited to
ASA I	A normal healthy patient	Healthy (no acute or chronic disease), normal body mass index percentile for age.
ASA II	A patient with mild systemic disease	Asymptomatic congenital cardiac disease, well-controlled dysrhythmias, asthma without exacerbation, well-controlled epilepsy, non–insulin-dependent diabetes mellitus, abnormal body mass index percentile for age, mild/moderate obstructive sleep apnea, oncologic state in remission, autism with mild limitations.
ASA III	A patient with severe systemic disease	Uncorrected congenital cardiac abnormality, asthma with exacerbation, poorly controlled epilepsy, insulin-dependent diabetes mellitus, morbid obesity, malnutrition, severe obstructive sleep apnea, oncologic state, renal failure, muscular dystrophy, cystic fibrosis, history of organ transplantation, brain/spinal cord malformation, symptomatic hydrocephalus, premature infant post-conceptual age <60 wk, autism with severe limitations, metabolic disease, difficult airway, long-term parenteral nutrition, full term infants <6 wks of age
ASA IV	A patient with severe systemic disease that is a constant threat to life	Symptomatic congenital cardiac abnormality, congestive heart failure, active sequelae of prematurity, acute hypoxic–ischemic encephalopathy, shock, sepsis, disseminated intravascular coagulation, automatic implantable cardioverter-defibrillator, ventilator dependence, endocrinopathy, severe trauma, severe respiratory distress, advanced oncologic state.
ASA V	A moribund patient who is not expected to survive without the operation	Massive trauma, intracranial hemorrhage with mass effect, patient requiring extracorporeal membrane oxygenation, respiratory failure or arrest, malignant hypertension, decompensated congestive heart failure, hepatic encephalopathy, ischemic bowel or multiple organ/system dysfunction.
ASA VI	A declared brain-dead patient whose organs are being removed for donor purposes	

5.3. Exclusion criteria

- History of thoracic surgery
- Patients with pulmonary pathology (atelectasis, pneumonia, pneumothorax, or pleural effusion) based on preoperative chest roentgenogram.
- Current respiratory infection with fever >38°C, purulent cough, yellowish nose discharge, or wheezing sound.
- Airway abnormality
- Increased intracranial or intraocular pressure
- Uncontrolled hypertension
- Uncontrolled seizure
- Allergy or contraindications for the study drugs
- Patients' refusal

5.4. Exit (drop out) from the Trial

- Patient withdrawal: Any participant has the right to withdraw their informed consent from the trial at any time. If a participant or parent of a participant chooses to withdraw their consent, they will exit the trial. The reason for the exit will be documented and reported. The participant will be asked to specify which aspects of the trial they are withdrawing their consent and participation from.
- MRI cancelation

6. Study design

6.1. Study Overview

This trial is a single center, parallel, double-blinded, randomized trial with a concealed 1:1 allocation. Pediatric patients scheduled for elective MRI scans under deep sedation will be randomly assigned to the propofol group or propofol–ketamine group (Figure 1). Participants (as well as their guardians), MRI technicians, radiologist, attending nurses in the PACU,

attending anesthesiologists, and outcome assessors will be blinded to treatment. This trial is initiated by the investigators and non-commercial.

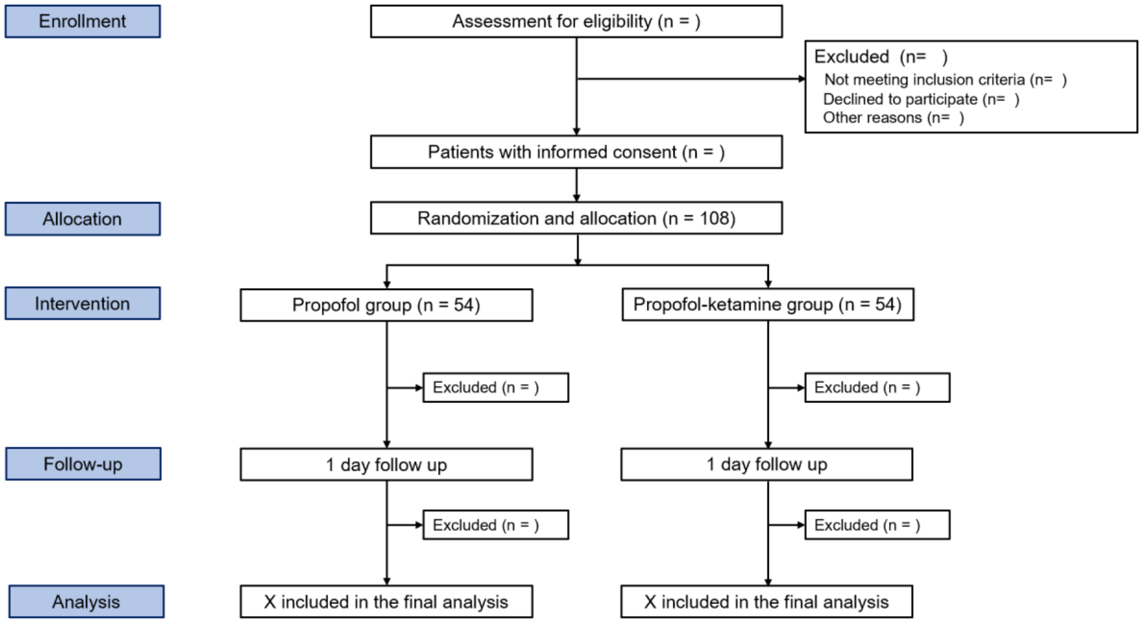

Figure 1. CONSORT Flow chart

6.2. Screening, Randomization and Blinding Procedure

6.2.1. Screening

Screening can be conducted either in the ward or in the outpatient unit. The main investigator will be responsible for screening all pediatric patients scheduled for elective MRI scans under deep sedation. A screening log will be complied whether they are eligible for inclusion or not. Informed consent will be obtained. Patients who meet the inclusion criteria but do not meet the exclusion criteria will be contacted by primary investigators one day before the MRI scan, or at the preanesthetic clinic on the day of MRI scan if it is performed in an outpatient setting. The patients will also be provided with an age-tailored brochure explaining the intervention.

6.2.2. Randomization

Randomization will be performed immediately before the induction of anesthesia. A web-based randomization program will be used (website, ‘sealedenvelope.com’) for this purpose. Enrolled subjects will be randomly allocated to either the propofol group or the propofol-ketamine group in a 1:1 ratio using block randomization with a block size of four.

6.2.3. Blinding

The group designation will remain blinded to the patients, their legal guardians, MRI technicians, radiologist, attending anesthesiologists, attending nurses in the PACU, and outcome assessors. All study drug will be administered to the patients using the equivalent volume and delivery rate.

- Patients: Patients are under deep sedation during the intervention.
- Legar guardians, MRI technicians and radiologists, attending anesthesiologists: The study drugs will be set up in an infusion pump system by assistant nurse before the patient enters the MRI suite. To prevent them from speculation about the randomized assignment, the study drugs for anesthesia induction will be prepared and administered as follows:

  1) Propofol group: Patients will receive 0.2 mL/kg of 1% propofol and 2 mL of 0.9% saline (placebo) followed by continuous infusions of propofol and 0.9% saline at rates of 200 µg/kg/min and 0.04 mL/kg/min, respectively.

  2) Propofol-ketamine group: Patients will receive 0.2 mL/kg of 0.5% propofol (a mixture of 1% propofol and 0.9% saline) and 1 mg/kg of diluted ketamine in 0.9% saline (total 2 mL) followed by continuous infusions of propofol and ketamine at rates of 100 and 20 µg/kg/min, respectively.

  The study drugs are identical in appearance between the two study groups and will be infused at the same rate, ensuring that group assignment remains unknown to the involved parties.

- Attending nurse in the PACU and outcome investigators: The study drugs will be stopped before the patient enters the PACU. They will remain blinded to the allocated group and will evaluate the postoperative outcomes.
- Anesthesia nurse: Prior to preparing the sedatives, the anesthesia nurse will open the envelope and prepared the study drug in a separate drug preparation space. They will be aware of the group allocation and have no other role in this study.

6.3. Study Intervention

6.3.1. Description of the Study Intervention

Before the induction of deep sedation, study participants will be randomly assigned in a 1:1 manner to one of the following anesthetic techniques:

- Propofol group: Propofol will be used for both induction and maintenance of anesthesia during the MRI scan.
    - Patients will receive an initial dose of 0.2 mL/kg of 1% propofol and 2 mL of 0.9% saline (placebo), followed by a continuous infusion of propofol and 0.9% saline at rates of 200 µg/kg/min and 0.04 mL/kg/min, respectively.
- Propofol-ketamine group: Both propofol and ketamine will be used for the induction and maintenance of anesthesia during the MRI scan.
    - Patients will receive an initial dose of 0.2 mL/kg of 0.5% propofol (a mixture of 1% propofol and 0.9% saline) and 1 mg/kg of diluted ketamine in 0.9% saline (total 2 mL), followed by a continuous infusion of propofol and ketamine at rates of 100 and 20 µg/kg/min, respectively.

In cases where sedation is not induced with the initial bolus doses of study drugs, patients will be administered an additional 1 mg/kg of propofol every one minute until they become unconscious.

6.3.2. Duration of Intervention

The assigned anesthetic strategy will be applied during anesthesia for MRI scan.

Final follow-up: 24 hours after MRI scan

6.3.3. Anesthesia, Surgery, and Postoperative Management

All participating patients, regardless of the study arm to which they are randomized, will be monitored and managed according to the general standard of care practices aimed at maintaining optimal conditions. Both intraoperative and postoperative management (unrelated to anesthetic management) will be determined by the attending anesthesiologists, following the established protocols at Samsung Medical Center. However, to ensure a high standard of anesthetic management, common strategies have been established:

1) During procedural sedation

- Intraoperative monitoring includes electrocardiography, non-invasive blood pressure, pulse oximetry, and end-tidal capnography measurements.
- Anesthesia will be maintained using either propofol or propofol-ketamine, respectively. Propofol will be titrated to maintain deep level of sedation
- To secure the airway, patients will be placed in a neck-extension position using a shoulder roll, unless there is contraindication for this position. Spontaneous ventilation will be maintained during the MRI scan, and oxygen will be supplied via nasal prong with a flow rate of 2 L / min. Oxygen saturation will be maintained above 95%. If oxygen desaturation occurs, airway intervention will be applied to treat sedation-induced respiratory depression: mild prodding, jaw thrust, reduction of study drug infusion rate, Guedel airway insertion, bag-mask-assisted ventilation, or intubation.

2) After MRI scan

- After the MRI scan, patients will be promptly transferred to PACU and stay there for one hour until they are fully alert and ready to ambulate.

- For patients who exhibit SpO2 <90%, oxygen will be administered via a facial mask of nasal prong.

6.4.1. Baseline Characteristics

These include,

- Age by birth date
- Height
- Weight
- Body mass index
- Sex
- ASA Physical Status

6.4.2. Procedure Characteristics

These include,

- Type of MRI scan
- Duration of MRI scanning
- Duration of anesthesia
- Total infused dose of anesthetics including propofol and ketamine
- Adverse events including tachycardia, bradycardia, hypertension, hypotension, desaturation, airway intervention, movement, interruption for scanning process.
- Quality of MRI scan

6.4.3. Postoperative variables (outcome variables)

These include,

- Lung ultrasound scores
- Diaphragm excursion
- Duration of PACU stay

- Time to emergence
- Emergence delirium
- Nurse satisfaction score
- Adverse events during the PACU stay: Dizziness, Nausea
- Parents satisfaction score
- Adverse events after the discharge from PACU until 24 hours after sedation: Dizziness, Drowsiness, respiratory complications, and others

7. Data Management

7.1. Data Handling and Record Keeping

The paper case record form (CRF) is used to record the relevant study data should not be altered. If case of any necessary correction, the modification made on the CRF should be signed with the date of modification. The completed original record form will be reviewed by principal investigator to ensure the accuracy, completeness, legibility, and timeliness of the reported data. All original records including consent forms, CRFs, and relevant correspondence, will be stored in a locked room within the hospital for a duration of 3 years, allowing inspection by relevant authorities if necessary. The trial database will be maintained for a period of 10 years and anonymized if requested for revision.

7.2. Quality Control and Quality Assurance

All investigators will be provided with sufficient information to participate in the trial. These documents include CRF, instructions for registration, checklists for inclusion/exclusion and randomization guidelines, and a protocol for medical treatment. The principal investigator holds the responsibility for verifying the accuracy and completeness of the reported data. Investigators will maintain adequate case histories of study participants, including accurate case report forms and source documentation.

8. Adverse Events

Detection, documentation and reporting of the following events will be the responsibility of the main investigator. Participants in the randomization cohorts will be monitored for severe adverse events (SAE) throughout their study surveillance period.

8.1 Definitions

SAEs are defined as one of the following conditions:

- Death during the period of protocol-defined surveillance
- Life-threatening event related to the intervention or significant disability/incapacity related to the intervention
- Event that requires hospitalization or prolongation of current hospitalization
- Event that results in persistent or significant disability or incapacity

8.2. Reporting of SAEs

All SAE cases will be recorded on the appropriate SAE CRF, reported to the study investigators and site institutional review boards (IRB) per their reporting guidelines. The determination of whether an SAE is related to the treatment will be assessed by the local investigator. The relatedness will be categorized as:

- Not related: The event is clearly related to other factors, such as the participant's clinical state, therapeutic interventions, or concomitant drugs administered to the participant
- Possibly related: There is a possible temporal relationship between the intervention and the event but it could have been caused by other factors
- Probably related: There is a plausible temporal relationship between the intervention and the event and the event is not reasonably explained by other factors.

The main investigator is required to follow each participant with an unexpected SAE until resolution of symptoms. The date of resolution (or when the event is deemed stable/chronic) will be noted on the appropriate case report form.

9. Ethical and Regulatory Considerations

This clinical trial will be conducted in accordance with the principles laid down by the 18th World Medical Assembly (Helsinki, 1964) and all applicable amendments laid down by the World Medical Assemblies, and the ICH guidelines for Good Clinical Practice (GCP). This clinical trial will be conducted in compliance with all national laws and regulations as well as any applicable guidelines.

9.1. Informed Consent

It is the investigator's responsibility to obtain informed consent from every study participant and their legal guardians through a dated and signed informed consent form before conducting any study-related procedures. The investigator, in accordance with applicable regulatory requirements, or a designated representative under the investigator's supervision, will comprehensively inform the patient or their legal guardians about all aspects of the clinical trial. All participants will receive full information about the study in terms they can understand. Prior to a patient's participation in the clinical trial, they must sign the written Informed Consent Form. It will be clearly communicated to the patient that they have the right to withdraw from the study at any time without providing reasons and that their decision to do so will not result in any disadvantage. Any Informed Consent will be included in the Investigator's file and retained along with it. A copy of the signed and dated written Informed Consent Form will be provided to the patient.

9.2. Independent Ethics Committee Approval

Prior to the initiation of the trial, the protocol, all informed consent forms, and any materials intended for prospective patients will be submitted to the appropriate IRB for approval

9.3. Responsibilities of the Investigator(s)

The investigator undertakes to perform the clinical trial in accordance with this clinical trial protocol, the current International Conference on Harmonization Guideline for GCP and the applicable regulatory requirements. The investigator ensures compliance with all procedures required by the clinical trial protocol and with all study required procedures. The investigator agrees to provide all information requested in the CRF in an accurate and legible manner.

9.4. Modification of the Protocol

Any protocol modification which may have impact on the conduct of this study, potential benefit of the patient or may affect patient safety, including changes of study objectives, study design, patient population, sample sizes, study procedures, or significant administrative aspects will require a formal amendment to the protocol. Such amendment will be agreed upon by principal investigator and approved by the Ethics Committee/IRB before implementation. Administrative changes of the protocol including minor corrections and/or clarifications that have no effect on the conduction of the study will be agreed upon by principal investigator. The Ethics Committee/IRB may be notified of administrative changes at the discretion of the principal investigator.

10. Statistical Analysis Plan

10.1. Randomization & allocation concealment

A designated study assistant will utilize “sealedenvelope.com” for the generation of random sheet, which will be prepared sequentially numbered, opaque, and sealed envelopes. These envelopes will be stored in a predetermined location with restricted access. The allocation sequence will remain concealed from the researcher responsible for recruiting participants. Only after the enrolled patient’s and other necessary information is written on the appropriate envelopes, the anesthesia nurse will open the envelope and prepare the study drug, who otherwise will have any involvement in this study.

10.2. Data collection/management

- Patient's baseline, procedural, and post-procedural data will be collected by investigators who is aware of the group allocation.
- Lung and diaphragm sonography will be performed by two experienced investigators who is blinded to the group allocation, using a handheld ultrasound system (VScan AirTM, GE Healthcare, Illinois, USA).
- Data will be entered to predetermined CRF and final analysis will be conducted when all the participants finish one day follow up. No interim analysis is planned.

10.3. Statistical Hypothesis

10.3.1. The primary statistical hypothesis is as follows:

Primary outcome: the incidence of lung atelectasis on lung ultrasonography at the end of sedation.

Atelectasis will be defined as a juxtapleural C score greater than 1 in more than one region based on the lung ultrasound examination.

Null hypothesis: the incidence of lung atelectasis of pediatric patients who underwent MRI scan with deep sedation are not different between those who randomized to the propofol group (p1) and propofol-ketamine group (p0); p1=p0
Alternative hypothesis: p1≠p0, two-tailed

10.3.2. The secondary statistical hypothesis is as follows:

10.3.2.1. Secondary outcome: Total lung score, diaphragm excursion, movement event, interruption for scanning process, quality of MRI scan, time to emergence, Duration of PACU stay, emergence delirium, nurse satisfaction, parent satisfaction, and respiratory complications within 24 hours after sedation.

Total lung score will be calculated as the sum of the C and B scores for seven lung regions.

Diaphragm excursion will be measured as the perpendicular distance between the upper border of the liver or spleen at the end of expiration and inspiration.[8] The diaphragm excursion values will be presented as the average excursion depth on each side.

Null hypothesis: The incidence of each of secondary outcome in pediatric patients who underwent MRI scan with deep sedation lung resection surgery are not different between those who randomized to the propofol group (p1) and propofol-ketamine group (p0); p1=p0

Alternative hypothesis: p1≠p0, two-tailed.

10.4. Sample Size Calculation

We calculated the sample size based on primary outcome, which is the incidence of atelectasis. Previous research reported that the incidence of atelectasis was 82% in children who underwent MRI scans under propofol sedation.[11] We anticipate that the incidence of atelectasis in the propofol-ketamine group to be reduced by 35% compared with the propofol group. The required sample size was determined to be 47, with a power of 85% and an alpha of 5%. Assuming a dropout rate of 10%, the study sample size was set at a total of 108 participants, with 54 participants in each group.

10.5. Statistical Methods

Data analysis will be performed using a “intention-to-treat” analysis, which means all randomized participants will be included in the primary analysis, except for those who meet dropout criteria, regardless of protocol adherence.

Data analysis will be executed according to a pre-established Statistical Analysis Plan. All statistical analyses will be performed by a team of statistics analysts utilizing MedCalc 19.5.6 (MedCalc Software Ltd., Ostend, Belgium), SPSS 27.0, or R software (version 4.2.2).

10.5.1. Primary outcome

The incidence of atelectasis: Categorical data will be presented as frequencies with percentages and compared using the chi-square test or Fisher's exact test. If the baseline and procedural characteristics demonstrate imbalance between the two study groups, multivariable logistic regressions will be conducted to adjust for the effect of imbalanced variables.

10.5.2. Secondary outcomes

10.5.2.1. Total lung score, diaphragm excursion, quality of MRI scan, time to emergence, Duration of PACU stay, and nurse satisfaction: Continuous variables will be analyzed using a two-sample t-test or Mann-Whitney test, as appropriate.

10.5.2.2. Postoperative complications

Movement event, interruption for scanning process, emergence delirium, parent satisfaction, and respiratory complications within 24 hours after sedation: Categorical data will be analyzed using the Chi-square test or Fisher's exact test, as appropriate.

10.5.3. Baseline characteristics

For continuous variables, median with interquartile range (IQR) (Q1-Q3) or means with standard deviation will be presented as appropriate based on normality assumption for continuous variables. For categorical variables, frequencies with percentages will be described. Group comparison for baseline characteristics will be performed using two-sample t-test or Mann-Whitney test as appropriate for continuous variables, and Chi-square test or Fisher's exact test for categorical variables.

11. Funding

The trial will be funded by a Samsung Medical Center grant.

12. Schedule of Events

	Screening	Preprocedural	Procedural	Post-procedural
	within 48 hours of admission	within 24 hours of admission	day 0	24 hours after sedation
**Eligibility screen**	√			
**Informed consent**		√		
**Baseline characteristics**		√		
**Randomization**		√		
**Intervention applied**			√	
**Anesthesia details**			√	
**Sedation data collection**			√	
**Lung sonography**			√	
**Postoperative data collection**			√	√
**Secondary outcome data collection including telephone interview**			√	√

13. Timeline

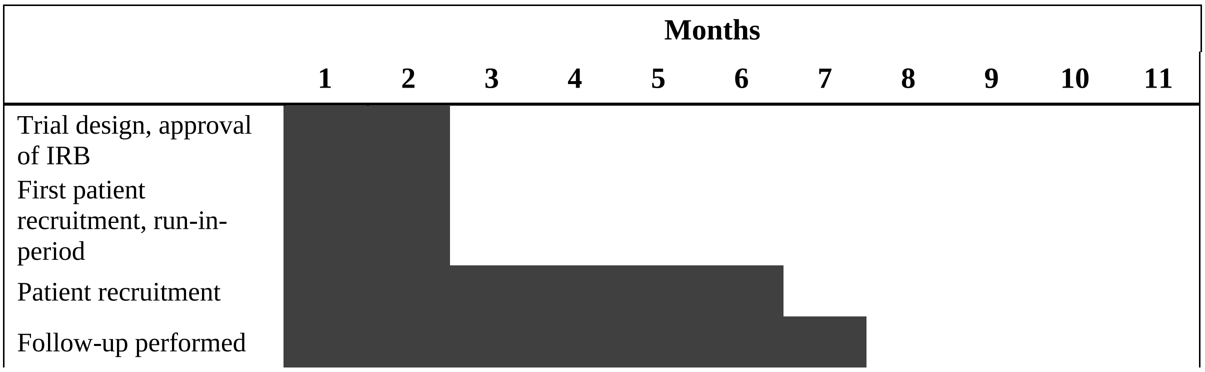

Data analysis	
Presentation of results	

14. Trial Participants

14.1. Participating Site

Samsung Medical Center, Sungkyunkwan University School of Medicine
Department of Anesthesiology and Pain Medicine
81 Irwon-Ro Gangnam-gu. Seoul, Republic of Korea (06351)

14.2. Principal investigator and research physician

Principal investigator and research physician will be responsible for the design and conduct of this study. They will prepare and revise the protocol, investigators' brochure, and CRF. They will also be members of steering committee meetings. Their responsibilities include the identification and recruitment of study patients, collaboration with the principal investigators, data collection, and completion of CRFs, along with follow-up of study patients. They will review the progress of study and if necessary, agree to amendments of the protocol or investigators' brochure while ensuring adherence to the study protocol and investigators' brochure. Additionally, they will oversee the publication of study reports.

Ji Seon Jeong MD, PhD	Samsung Medical Centre,
Yu Jeong Bang MD	Sungkyunkwan University School of Medicine,
Jeayoun Kim MD	Seoul, South Korea.

14.4 Investigators

Investigators will plan and oversee the conduct of the study, as well as collect and verify study data. They will provide guidance and advice for principal investigators and promptly report serious unexpected suspected adverse events to principal investigators and ethics committee.

Nam-Su Gil, MD, PhD	Samsung Medical Centre,

Woo Seog Sim, MD, PhD	Sungkyunkwan University School of Medicine, Seoul, South Korea.
Hyun Joo Ahn, MD, PhD	
Mi-Hye Park, MD, PhD	
Sangmin Maria Lee, MD, PhD	
Dong-Jae Kim, MD	

14.5. End-point adjudication committee

The endpoint adjudication committee will conduct a thorough review of all available laboratory and clinical data to resolve any uncertainties related to the trial outcomes. They will remain blinded to the assigned study group. Confirmation reports of all detected outcomes will be de-identified and submitted to the end-point adjudication committee. The adjudication committee will consist of a panel of two blinded experts.

14.6. Data monitoring committee

Anesthetics used in this study are routinely administered during anesthesia. The probability and magnitude of harm or discomfort anticipated in the research are not greater in and of themselves than those ordinarily encountered in regular perioperative practice except for the risk of randomization. In addition, we will not perform interim analyses. Therefore, we will not establish a data monitoring committee.

14.8. Statistical experts

Statistical experts of Biomedical Statistics Center, Research Institute for Future Medicine, Samsung Medical Center will independently review data and perform statistical analysis. They also help with data cleaning, statistical analysis, and data visualization.

Reference

1. Deen J, Vandevivere Y, Van de Putte P. Challenges in the anesthetic management of ambulatory patients in the MRI suites. *Curr Opin Anaesthesiol*. 2017;30(6):670-675. doi:10.1097/aco.0000000000000513

2. Mallory MD, Travers C, Cravero JP, Kamat PP, Tsze D, Hertzog JH. Pediatric Sedation/Anesthesia for MRI: Results From the Pediatric Sedation Research Consortium. *J Magn Reson Imaging*. 2023;57(4):1106-1113. doi:10.1002/jmri.28392

3. Ko E, Yoo KY, Lim CH, Jun S, Lee K, Kim YH. Is atelectasis related to the development of postoperative pneumonia? a retrospective single center study. *BMC Anesthesiology*. 2023;23(1):77. doi:10.1186/s12871-023-02020-4

4. Lagier D, Zeng C, Fernandez-Bustamante A, Vidal Melo MF. Perioperative Pulmonary Atelectasis: Part II. Clinical Implications. *Anesthesiology*. 2022;136(1):206-236. doi:10.1097/ALN.0000000000004009

5. Khurmi N, Patel P, Kraus M, Trentman T. Pharmacologic Considerations for Pediatric Sedation and Anesthesia Outside the Operating Room: A Review for Anesthesia and Non-Anesthesia Providers. *Paediatr Drugs*. 2017;19(5):435-446. doi:10.1007/s40272-017-0241-5

6. Song IK, Jang YE, Lee JH, Kim EH, Yoo S, Kim HS, Kim JT. Effect of different fraction of inspired oxygen on development of atelectasis in mechanically ventilated children: A randomized controlled trial. *Paediatr Anaesth*. 2019;29(10):1033-1039. doi:10.1111/pan.13718

7. Acosta CM, Maidana GA, Jacovitti D, et al. Accuracy of transthoracic lung ultrasound for diagnosing anesthesia-induced atelectasis in children. *Anesthesiology*. 2014;120(6):1370-1379. doi:10.1097/ALN.0000000000000231

8. El-Halaby H, Abdel-Hady H, Alsawah G, Abdelrahman A, El-Tahan H. Sonographic Evaluation of Diaphragmatic Excursion and Thickness in Healthy Infants and Children. *J Ultrasound Med*. 2016;35(1):167-175. doi:10.7863/ultra.15.01082

9. Sikich N, Lerman J. Development and psychometric evaluation of the pediatric anesthesia emergence delirium scale. *Anesthesiology*. 2004;100(5):1138-1145. doi:10.1097/00000542-200405000-00015

10. Leahy I, Berry JG, Johnson CJ, Crofton C, Staffa SJ, Ferrari L. Does the Current American Society of Anesthesiologists Physical Status Classification Represent the Chronic Disease Burden in Children Undergoing General Anesthesia? *Anesthesia & Analgesia*. 2019;129(4):1175-1180. doi:10.1213/ane.0000000000003911

11. Lutterbey G, Wattjes MP, Doerr D, Fischer NJ, Gieseke Jr J, Schild HHJPA. Atelectasis in children undergoing either propofol infusion or positive pressure ventilation anesthesia for magnetic resonance imaging. 2007;17(2):121-125.
