# Supplementary material for: Pulmonary Atelectasis After Sedation With Propofol vs Propofol-Ketamine for Magnetic Resonance Imaging in Children: A Randomized Clinical Trial
Source: JAMA Netw Open. 2024 Nov 1;7(11):e2433029. doi: 10.1001/jamanetworkopen.2024.33029 (PMC11530935; doi:10.1001/jamanetworkopen.2024.33029)
Supplement: Supplement 2. — Data Sharing Statement [file jamanetwopen-e2433029-s002.pdf]

# Data Sharing Statement

Bang. Pulmonary Atelectasis After Sedation With Propofol vs Propofol-Ketamine for Magnetic Resonance Imaging in Children. *JAMA Netw Open*. Published September 25, 2024. doi:10.1001/jamanetworkopen.2024.33029

**Data**
**Data available:** Yes
**Data types:** Deidentified participant data
**How to access data:** The authors confirm that the data supporting the findings of this study are available within the article. Individual participant and additional data are available from the corresponding author, JSJ, upon request (wltjs78@skku.edu; jiseon78.jeong@samsung.com).
**When available:** With publication

**Supporting Documents**
**Document types:** None

**Additional Information**
**Who can access the data:** Researchers whose proposed use of the data has been approved
**Types of analyses:** Meta-analysis
**Mechanisms of data availability:** After apporval of a proposal from the principal investigator of this trial and institutional review board of Samsung Medical Center.
